# Cocreating an Automated mHealth Apps Systematic Review Process With Generative AI: Design Science Research Approach

**DOI:** 10.2196/48949

**Published:** 2024-02-12

**Authors:** Guido Giunti, Colin P Doherty

**Affiliations:** 1 Academic Unit of Neurology School of Medicine Trinity College Dublin Dublin Ireland; 2 Research Unit of Health Sciences and Technology Faculty of Medicine University of Oulu Oulu Finland; 3 FutureNeuro SFI Research Centre Royal College of Surgeons in Ireland Dublin Ireland; 4 Department of Neurology St James Hospital Dublin Ireland

**Keywords:** generative artificial intelligence, mHealth, ChatGPT, evidence-base, apps, qualitative study, design science research, eHealth, mobile device, AI, language model, mHealth intervention, generative AI, AI tool, software code, systematic review, language model

## Abstract

**Background:**

The use of mobile devices for delivering health-related services (mobile health [mHealth]) has rapidly increased, leading to a demand for summarizing the state of the art and practice through systematic reviews. However, the systematic review process is a resource-intensive and time-consuming process. Generative artificial intelligence (AI) has emerged as a potential solution to automate tedious tasks.

**Objective:**

This study aimed to explore the feasibility of using generative AI tools to automate time-consuming and resource-intensive tasks in a systematic review process and assess the scope and limitations of using such tools.

**Methods:**

We used the design science research methodology. The solution proposed is to use cocreation with a generative AI, such as ChatGPT, to produce software code that automates the process of conducting systematic reviews.

**Results:**

A triggering prompt was generated, and assistance from the generative AI was used to guide the steps toward developing, executing, and debugging a Python script. Errors in code were solved through conversational exchange with ChatGPT, and a tentative script was created. The code pulled the mHealth solutions from the Google Play Store and searched their descriptions for keywords that hinted toward evidence base. The results were exported to a CSV file, which was compared to the initial outputs of other similar systematic review processes.

**Conclusions:**

This study demonstrates the potential of using generative AI to automate the time-consuming process of conducting systematic reviews of mHealth apps. This approach could be particularly useful for researchers with limited coding skills. However, the study has limitations related to the design science research methodology, subjectivity bias, and the quality of the search results used to train the language model.

## Introduction

The delivery of health-related services through the use of mobile devices (mHealth) [1] has been growing at a tremendous pace. A decade ago, in the first “era of mHealth,” the literature surrounding mHealth called for the generation of evidence demonstrating the impact of mHealth solutions on health system processes and patient outcomes [2]. In 2013, Labrique et al [2] conducted a preliminary search on the US federal clinical trials database (ClinicalTrials.gov) and had to combine the keywords “mHealth,” “mobile,” and “cell AND phone” to obtain 1678 studies and their results. Today, that same number can be obtained using “mHealth” alone as a keyword. As the need for mHealth evidence has grown, so too has the necessity for summarizing both the state of the art and the practice.

Systematic reviews seek to collect and combine relevant evidence within the specific scope of a research question while also striving to minimize bias [3,4]. In PubMed alone, the number of systematic reviews published on digital health–related topics has increased a hundredfold in the last 10 years. In fact, the pace at which the mHealth field is developing for certain conditions like breast cancer is such that systematic reviews can be found every 2 or 3 years [5-9]. The systematic review process, however, is a time- and resource-intensive process, reportedly requiring a median of 5 researchers and approximately 40 weeks of work to reach submission [10-12].

The emergence of generative AI has been seen as a breakthrough in the field of automation. With the ability to generate content such as text, images, and even music, AI has been reported as a potential solution to tedious time-consuming and labor-intensive tasks [13]. For instance, generative AI can be used to automatically generate product descriptions, news articles, or even code [14]. By eliminating the need for human intervention, generative AI can free up valuable time and resources for more complex tasks, thereby improving efficiency and accuracy. ChatGPT, a natural language processing model with a capacity of 175 billion parameters, has been trained on extensive amounts of data and is designed to produce human-like responses to user inputs. Since its release in November 2022, ChatGPT has received significant attention from media and academia alike, provoking ethical discussions on scientific authorship [15,16], attempting to pass medical license and specialist examinations [17-19], and even designing medical education curricula [20].

The objective of this study was to explore the feasibility of using generative AI tools to automate time-consuming and resource-intensive tasks in a systematic review process and assess the scope and limitations of using such tools.

## Methods

### Study Design

**Figure 1:**
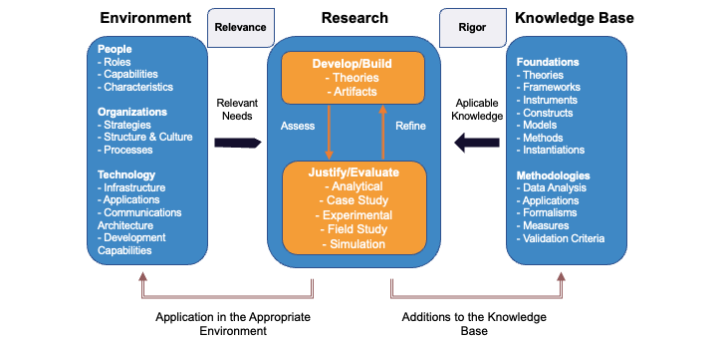
Design science research overview, adapted from Hevner 2004 [22].

This study uses a design science research (DSR) methodology. DSR is a problem-solving paradigm that seeks to enhance human knowledge via the creation of innovative artifacts [21]. DSR commonly involves the identification of a problem or opportunity, followed by the development, implementation, and evaluation of a solution. In DSR, as well as in action research, the process happens within an organization that provides context and that would be changed as a result of the use of the artifact [21]. An overview of the process adapted from Hevner [22] can be seen in Figure 1.

### Problem Definition

The problem to which DSR was applied was the time-consuming and resource-intensive process of conducting systematic reviews of mHealth applications.

### Organizational Context

The organizational context consisted of the More Stamina team of researchers, software developers, and health care professionals, working collaboratively within the host research institutions (ie, the University of Oulu and Trinity College Dublin).

The More Stamina project aims to create an evidence-driven gamified mHealth solution for people with multiple sclerosis (MS), where each step of the development follows a scientific process, as follows: MS needs as well as barriers and facilitators were explored through qualitative studies [23]; the state of the practice for MS apps was systematically reviewed [24,25]; user-centered design was used to create “MS personas” [23]; cocreation sessions took place to produce solution concepts [26]; the design, prototyping, and initial usability testing were described [27]; early health technology assessment was used to guide software development [28]; patient representatives were involved throughout the project [29]; and user testing and feasibility studies were ongoing in a multicenter study [30].

A script using the software application for audience targeting called 42matters [31] was used in the past to extract information from different app stores. The script is no longer functional, and person-hours from the software development team were not able to be dedicated to this task.

### Background Studies

The research plans and outlines from previous studies, where systematic review methodologies were used to identify, select, collect, and analyze features and content of mHealth apps [6,24,25], served as models for our study. In those studies, a search strategy was defined, using relevant main keywords for each condition. App stores were searched, taking steps to ensure that no previous search history or cookies influenced results. Screening took place based on mHealth applications’ titles, descriptions, and metadata.

### Solution

The solution was to apply a cocreation process with a generative AI (ie, ChatGPT 3.5, as of June 2023) to produce software code that automated the process for conducting systematic reviews.

### Cocreation Goal

The goal of the cocreation process was to use ChatGPT as a design and development partner for the automation process. The generative AI was to be interacted with as if it were a valid interloper who was more technologically skilled than the user and was guiding them through the process over text messages.

### Development and Implementation

Development and implementation of the automated process happened through iterative and continuous conversations with the generative AI by one of the authors (GG). GG is a primary care physician with over a decade of experience leading digital health software design and development. Table 1 provides an overview of his digital skills background using the European Qualifications Framework and with a self-assessment score from 1 to 10 to describe his competency level. Regardless of the skill level, the development cycle was to be conducted as if no coding skill was present on the part of the user.

**Table 1:** Digital skills background.

Competency	Level	Experience	Self-assessment score (of 10)
Scrum master	Certified Scrum Master	Agile methodologies and team management	7
Product owner	Certified Scrum Product Owner	Product road mapping and stakeholder management	8
Game design	Intermediate	Game mechanics, storytelling, and level design	7
Web design	Advanced	User experience and user interface design and responsive design	8
JavaScript	Beginner	Front-end development	5
HTML5	Intermediate	Front-end development	7
PHP: Hypertext Preprocessor	Beginner	Front-end and back-end development	4

### Evaluation

The results were evaluated for their effectiveness and efficiency in replicating the initial steps of the background studies. The measurements were considered with respect to the amount of time required to generate a spreadsheet containing the necessary information for human reviewers to start the systematic review process. The output was compared to the output generated by the previous script, which required further processing of the data.

### Ethical Considerations

No ethics board review is needed as the work does not use patient data or involve human participants.

## Results

The following section reports the overall design, development, and implementation process as well as the evaluation results and highlights of the experience. Prompts provided by GG and generative AI are tagged accordingly.

### Cocreation Process

ChatGPT requires prompts to trigger interaction. For this purpose, a lay language description of the desired outcome was engineered by GG (triggering prompt), as follows:

I need a script that crawls the Google Play Store for apps that target people with multiple sclerosis. The script should generate a spreadsheet file that contains name, links, description and other metadata, as well as have a specific column to highlight if the description mentions any medical or clinical evidence. The script should then search Pubmed database looking for each app listed on the spreadsheet, and create a separate spreadsheet relating the apps with any scientific publication that mentions them in Pubmed.

GG

**Figure 2:**
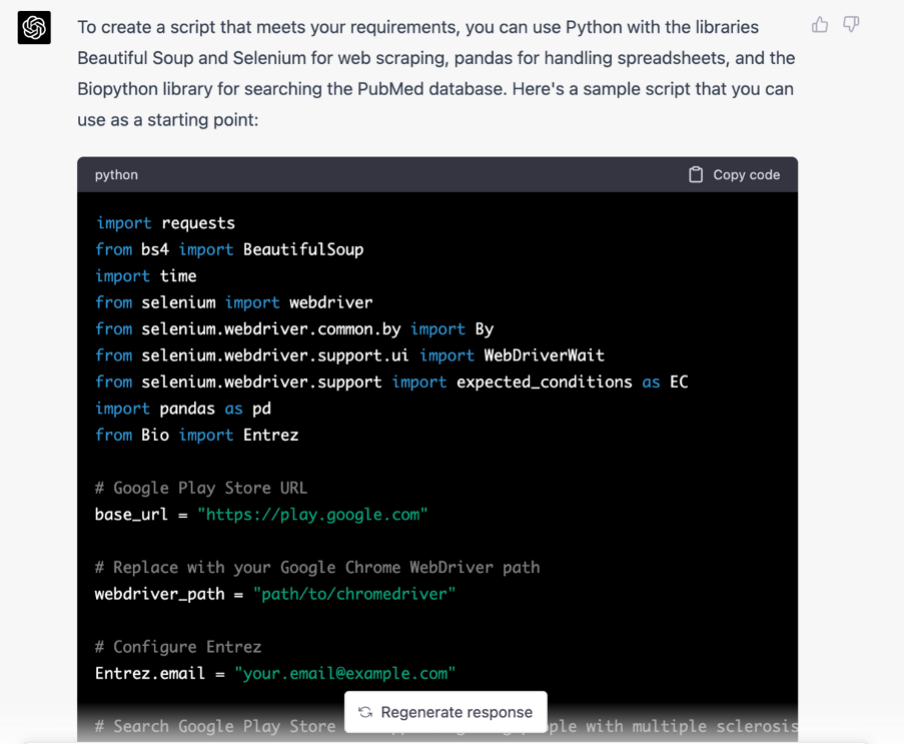
ChatGPT's first response to the initial prompt.

The generative AI’s response was to provide a sample script in Python that was suggested as a starting point, as shown in Figure 2.

Following the premise of the cocreation goal, ChatGPT was asked (by GG) “I am new to Python, how do I run a Python script?” to which the response was the following:

To run a Python script, you need to follow these steps:

1. Install Python on your computer, if it's not already installed. You can download Python from the official website: https://www.python.org/downloads/.

2. Open a text editor (e.g. Notepad, Sublime Text, Atom) and create a new file.

3. Write your Python code in the file.

4. Save the file with a .py extension (e.g. “my_script.py”).

5. Open a command prompt or terminal and navigate to the directory where your Python script is saved.

6. Type “python” followed by the name of your script and press enter. For example, if your script is named “my_script.py”, you would type “python my_script.py” and press enter.

7. Your Python script will now run, and you should see any output or results in the command prompt or terminal.

Note: If you're using an Integrated Development Environment (IDE) like PyCharm or Visual Studio Code, you can run your script directly from the IDE by clicking on the “Run” button or pressing a keyboard shortcut.

ChatGPT

ChatGPT provided step-by-step guidance for the installation and setup of Visual Studio Code, along with the required libraries and dependencies. After the development environment was ready, the suggested Python script was copied and pasted to check its functionality. The code produced several errors, which were fed back to ChatGPT by GG, as follows:

I am getting “ModuleNotFoundError: No module named 'requests'”

Now it says “ModuleNotFoundError: No module named 'bs4'”

ChatGPT stated that the “requests” library was imported but not used, so it should be removed. Subsequent error messages were troubleshot with the AI. At times, the script would stop responding due to a time-out error or issues with the output. To make the debugging process easier to follow and understand, ChatGPT was prompted by GG to do the following:

add error messages that explain what went wrong.

add indicators for each step that it is working.

could you add a progress bar indicator to each step?

I would like the responses to be colored.

**Figure 3:**
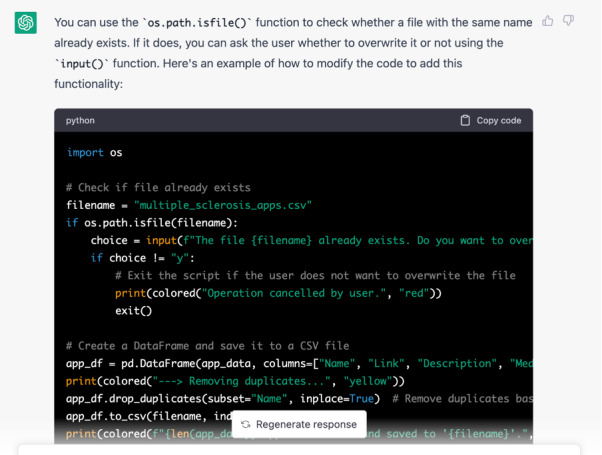
Added libraries and code for checking existing files.

Upon review, it was clear that some error caused the output files to be overwritten. The author requested the following: “how would you add a condition to check whether a file with the same name already exists and if it does ask whether to overwrite it?” The AI added the pertinent libraries to the script and produced an input prompt for the user (Figure 3).

The triggering prompt expected the script to not only be searching app stores for matches with “multiple sclerosis” but also to cross-reference them with scientific publications found in PubMed. As described in the Background Studies section of this paper, this process was done by looking for keywords present in the app description. As it were, the script was designed to only search for “multiple sclerosis” and crashed when it reached the PubMed phase. ChatGPT diagnosed a time-out error due to the number of publications and provided a potential solution to improve the indicator bars:

The progress bar for PubMed search may not be moving because the total value for it is set to 3600, which is the number of seconds in an hour. This is the unit being used for the progress bar, not the total number of items to be searched. To fix this, you should update the total value for the progress bar to be the total number of apps being searched, which is the length of the app_df dataframe multiplied by the maximum number of PubMed IDs to be retrieved for each app (100 in this case). You can update the progress bar as follows…

ChatGPT

After these issues were sorted and the script could properly fetch PubMed results, more keywords were entered into the script by prompting ChatGPT with the following:

how would you make it so that the items in the medical_keywords list are taken from a csv file called “keywords.csv”?

GG

**Figure 4:**
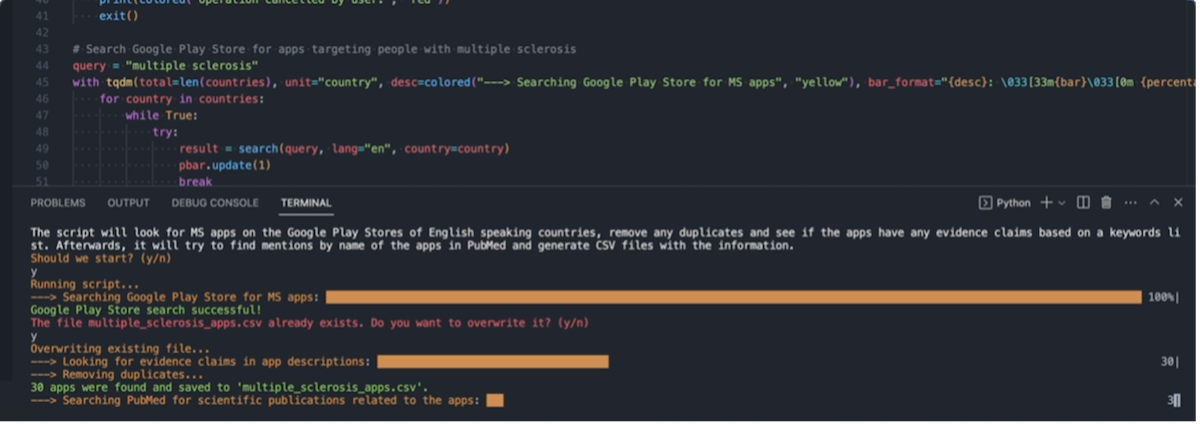
Screenshot of the final script running.

A screenshot of the final Python script running can be seen in Figure 4.

### Evaluation

As explained in the Background Studies section of this paper, app data extraction from the Google Play Store resulted in a spreadsheet file that contained the mHealth app’s name, store link, app description, developer’s name, developers’ URL, price, number of downloads, and app rating. During the screening phase of the studies, the research team read the apps’ descriptions and flagged those that contained keywords or sentences suggestive of the evidence base for in-depth review.

The ChatGPT-generated code resulted in a CSV file that contained the app’s name, store link, app description, and a column titled “Medical Evidence.” There were no columns containing metadata, and the Medical Evidence column only contained “Yes” or “No,” accordingly. Closer inspection revealed that the script was searching for a full match on the apps’ titles in PubMed results. The resulting document was useful as an intermediate outcome but was deemed unsuitable as a final output. The overall cocreation process had a total duration of 4 hours and 39 minutes, providing a working script version available on GitHub [32].

Using the results from the ChatGPT-generated script to fully automate the process would likely require further work refining the script, either by using the steps of the background studies to base the script or by providing clearer starting prompts for the generative AI. However, leveraging this approach as a means to advance work when the software developing team was otherwise engaged was useful.

### Highlights

Some highlights of this study are as follows:

- The overall cocreation process exercise had a total duration of 4 hours and 39 minutes.
- There were several misunderstandings during the interactions, not unlike the challenges one might encounter when messaging a more experienced coder.
- Structured thinking ahead of time reduced the number of misunderstandings.
- No knowledge of Python scripting was required by the author.
- The resulting output was useful to continue a systematic review but not sufficient to replace the final outputs.

## Discussion

### Principal Results

This study is the first to describe the cocreation process with a generative AI in developing an automated script for conducting a systematic review of mHealth apps. The study provides insights into the potential of using this kind of AI tools for researchers with little to no coding skills, and it identifies an innovative way of approaching a research problem and facilitating interdisciplinary collaborations. This study also makes a methodological contribution, expanding knowledge as it uses DSR, an approach that is not commonly used in health care and health informatics [33].

### Comparison With Prior Work

The resource-intensive process and the burden that systematic reviews represent have been highlighted in the literature before. The use of multiple databases, such as MEDLINE, Embase, Cochrane Library, and Web of Science as well as clinical trial registries like ClinicalTrials.gov are common practices to increase results [34]. However, this tactic requires a lengthy deduplication process, involving long manual procedures, potentially introducing quality-affecting errors and biases [35-37]. In fact, automation attempts using AI models have been made in the past, with a focus on the deduplication problem, as seen in studies by Borissov et al [38] and Bramer et al [39].

Performing a systematic review is a common step in doctoral researchers’ studies [40,41], as a means of introducing the candidate to the topic. The use of generative AI to cocreate scripts like the one presented in this study could help automate the time-consuming process, allowing researchers to focus on other aspects of the research process.

The ethical implications of using generative AI models, such as ChatGPT, to generate scientific authorship have sparked discussions [15,16]. AI’s potential for assisting in academic research needs to be considered and weighed against the potential for its misuse. Although generative AI can assist in the development of a systematic review script, it is important to note that the final review still requires human oversight and input to not only assess the accuracy and relevance of the results but also ensure that the ethical principles have been followed.

Beyond research, there are wider implications for the use of generative AI in both medical education and the upskilling of the health care workforce. The need for more digital skills training for health care professionals is widely recognized [42], and other authors have further explored medical degree programs’ curricula to examine how AI is included [43,44]. A recent publication explored the specific competencies needed for the effective and ethical use of AI in health care [45]. Understanding basic knowledge of AI and its applications as well as how to integrate AI into the general workflow of different tasks ranked among the top 6 key competency domains.

The role of generative AI in evolving health care education is pivotal, especially as universities adapt to its challenges. Generative AI has the potential to streamline processes like systematic reviews and clinical information retrieval, thereby allowing health care professionals to focus more on patient-centered, empathetic care and the co-design of effective treatment outcomes.

### Limitations

The results of this study must be considered within its limitations. The DSR methodology was developed for this specific problem, which limits applicability in other contexts. In addition, subjectivity is a common bias present in DSR, which can make it difficult to establish the reliability and validity of the results. The main goal of DSR is to generate prescriptive knowledge, which provides guidelines on how to effectively design and implement solutions in the organizational context. However, as DSR focuses more on developing practical solutions rather than generating new theoretical insights, it was aligned with the goal of this study. DSR differs from traditional research paradigms by focusing more on creating and evaluating new solutions rather than on understanding existing phenomena. Further, while generative AI can assist in the development of a systematic review script, the result will be greatly affected by the training data used for the language model. Additionally, there may be limitations in the quality of the search results obtained from the previous studies, which only become apparent through automated processes.

### Conclusions

This study outlined the cocreation process of an automated script for systematic reviews of mHealth apps, using generative AI. The study shed light on the potential of such AI tools for researchers with limited coding abilities and highlighted a novel approach for addressing research problems and promoting interdisciplinary collaborations.

## Abbreviations

AI: artificial intelligence
DSR: design science research
mHealth: mobile health
MS: multiple sclerosis
